# PON-1 Activity and Plasma 8-Isoprostane Concentration in Patients with Angiographically Proven Coronary Artery Disease

**DOI:** 10.1155/2016/5136937

**Published:** 2015-11-30

**Authors:** Agnieszka Kuchta, Adrian Strzelecki, Agnieszka Ćwiklińska, Magdalena Totoń, Marcin Gruchała, Zbigniew Zdrojewski, Barbara Kortas-Stempak, Anna Gliwińska, Kamil Dąbkowski, Maciej Jankowski

**Affiliations:** ^1^Department of Clinical Chemistry, Medical University of Gdańsk, Marii Skłodowskiej-Curie 3a, 80-210 Gdansk, Poland; ^2^Chair and Clinic of Internal Medicine, Connective Tissue Diseases and Geriatrics, Medical University of Gdańsk, Marii Skłodowskiej-Curie 3a, 80-210 Gdansk, Poland; ^3^First Chair & Clinic of Cardiology, Medical University of Gdańsk, Marii Skłodowskiej-Curie 3a, 80-210 Gdansk, Poland

## Abstract

The aim of the study was to estimate association of the extent of angiographically proven coronary artery disease (CAD) with plasma 8-isoprostane F2 (8-iso-PGF2*α*) levels as a reliable marker of lipid peroxidation and serum activity of paraoxonase-1, which demonstrates the ability to protect against lipid oxidation. The study included 105 patients with angiographically documented CAD (CAD+) and 45 patients with negative results of coronary angiography (CAD−). Compared to the control group CAD+ patients were characterized by increased 8-iso-PGF2*α* levels (*P* = 0.007) and reduced activity of PON-1 towards paraoxon (PONase, *P* = 0.002) and phenyl acetate (AREase, *P* = 0.037). Univariate correlation analysis indicated that 8-iso-PGF2*α* concentrations were positively associated with the severity of CAD as evaluated by the Gensini score (*R* = 0.41, *P* < 0.001) while PONase activity (*R* = −0.26, *P* < 0.05) and AREase activity (*R* = −0.23, *P* < 0.05) were inversely correlated with CAD severity. PONase activity and 8-iso-PGF2*α* concentration remained independent determinant of atherosclerosis severity in multiple linear regression after adjusting for age, gender, smoking habits, hypertension, type 2 diabetes, statin therapy, and HDL-C and TAG concentration (*β* coefficients −0.267; *P* < 0.05 and 0.368; *P* < 0.001, resp.). The results suggest that PON-1 activity and 8-iso-PGF2*α* concentration are associated with the presence and extent of coronary stenosis and may be considered additional markers of coronary artery disease.

## 1. Introduction

The mechanisms of the onset and development of atherosclerosis are still not entirely resolved although oxidation of lipoproteins seems to be essential to this process [1, 2].

Various biomarkers of lipid peroxidation are recently of great interest not only for highlighting pathological mechanisms, but also for clinical applications as biomarkers. Among them isoprostanes, products of nonenzymatic lipid peroxidation, seem to be particularly valuable. Isoprostanes, specifically 8-iso-prostaglandin F2 (8-iso-PGF2*α*), are recently indicated as the most valid* in vivo* lipids peroxidation biomarkers [3, 4] which themselves exert proatherogenic function by means of their vasoconstrictive, platelet-activating, and mitogenic properties [5, 6].

The second biomarker with sustained interest of researchers is the high-density lipoprotein associated enzyme: paraoxonase-1 (PON-1). This enzyme hydrolyzes aromatic carboxylic acid esters, organophosphates, and oxidized phospholipids, simultaneously destroying biologically active lipids in mildly oxidized lipoproteins, thus protecting them against further oxidation [7, 8]. Several lines of evidence suggest that PON-1 has antioxidant and atheroprotective effects. Genetic deletion of PON-1 in animal models of atherosclerosis is associated with increased oxidation of low-density lipoproteins (LDLs), increased macrophage oxidative stress, and increased atherosclerotic lesion size [9–11]. Conversely, overexpression of human PON-1 in transgenic mice results in reduction of aortic lesion size and corresponding decreases in oxidized lipid-protein adduct levels [12, 13]. It has been shown that PON-1 activity is associated with accelerated atherosclerosis [14] but is also affected both by genetic polymorphism and by environmental factors including age, lifestyle, and pharmaceutical intervention [15, 16].

Classically, PON-1 activity in serum is named after the substrate used to monitor enzymatic function, namely, paraoxonase activity (using paraoxon as substrate) and arylesterase activity (using phenyl acetate as substrate). A previous report has shown that the phenotype distinguished on the basis of the paraoxonase-to-arylesterase ratio closely corresponds to a common PON-1 polymorphism: Q (Glutamine) or R (Arginine) at codon 192 [15, 17, 18].

The purpose of this study was to test whether PON-1 activity, assessed by its ability to hydrolyse paraoxon or phenyl acetate, as well as plasma 8-iso-PGF2*α* concentrations can be used as indicators for atherosclerotic processes in coronary arteries. For this purpose we analyzed the association of plasma 8-iso-PGF2*α* levels and paraoxonase (PONase) and arylesterase (AREase) activity with the extent and severity of angiographically proven coronary artery disease (CAD) assessed by Gensini score.

## 2. Methods

### 2.1. Patients

The study group consisted of 150 patients undergoing coronary angiography for suspected CAD at the Medical University of Gdańsk (Poland). All subjects were in stable condition. None of the subjects had sustained a myocardial infarction within 6 months prior to taking part in the study. Patients with acute coronary syndrome or hepatic or renal disorders were excluded. The study was approved by the Independent Ethics Committee of the Medical University of Gdańsk and all patients gave their informed consent.

### 2.2. Coronary Angiography

Coronary angiography was performed using the transradial or femoral approaches in all recruits. The severity and extent of coronary atherosclerosis were quantified for each patient using the Gensini score, an assessment with prognostic significance for predicting the incidence of death or other cardiovascular events [19]. The Gensini score was assigned according to a previously described protocol [20]. Patients were divided into two groups: those with CAD (Gensini score ≥1; CAD+) and those without (Gensini score = 0; CAD−) according to angiographic results.

### 2.3. Laboratory Measurements

Blood samples were obtained between 7 and 8 a.m. on the day of and prior to coronary angiography following an overnight fast. Samples (serum and plasma) were separated after collection by centrifugation at 1000 ×g for 15 min and stored at −80°C pending analysis.

Total cholesterol (TC) and triacylglycerols (TAG) were measured in serum using standard enzymatic colorimetric tests. High-density lipoprotein cholesterol (HDL-C) was determined following precipitation of apolipoprotein B containing lipoproteins; LDL cholesterol level (LDL-C) was calculated using the Friedewald formula.

8-Iso-PGF2*α* was analyzed in plasma using an enzyme immunoassay kit (Cayman Chemical Company, USA). Data are expressed in pg/mL. The intra- and interassay coefficients of variation were 7.6 and 8.8%, respectively.

Paraoxonase (PONase) and arylesterase (AREase) activity were measured in serum based on paraoxon and phenyl acetate hydrolysis, respectively, according to procedure described earlier [17, 21]. The intra- and interassay coefficients of variation were 4.5 and 6.7%, for the PONase activity assay, and 2.8 and 5.5% for the AREase activity assay, respectively.

### 2.4. Statistics

All statistical analyses were performed using STATISTICA software, version 10. The Shapiro-Wilk test was used to test the distribution of variables that followed a Gaussian pattern. Continuous variables were expressed as mean ± SD (standard deviation) or medians with 25th and 75th percentiles. Student's unpaired *t*-test or the Mann-Whitney *U* test was used to assess the differences between two groups. Pearson's chi-squared test was used to compare categorical variables. Univariate correlations were assessed using standardized Spearman coefficients. Skewed variables, like PONase and 8-iso-PGF2*α*, were log-transformed to normal distribution for multiple linear regression analysis. Multilinear regression was assessed using standardized *β* coefficients. *P* values below 0.05 were considered statistically significant.

## 3. Results

The result of coronary angiography confirmed atherosclerosis in 105 patients; 45 patients received a negative result.

Clinical characteristics of patients with angiographically proven coronary artery disease (CAD+) and patients with negative results from coronary angiography (CAD−) are shown in Table 1. The groups were matched for sex, age, BMI, smoking habit, preexisting hypertension, diabetes, and metabolic syndrome. Statins were being taken by 90% of patients with confirmed atherosclerosis and by 55% in the group with negative results of angiography. Compared to the CAD− group, CAD+ patients had significantly lower mean concentrations of total cholesterol by 14%, LDL cholesterol (LDL-C) by 19%, HDL cholesterol (HDL-C) by 15%, and Apo AI by 12%. Concentrations of triacylglycerols and Apo B were similar in both groups (Table 1).

**Figure 1:**
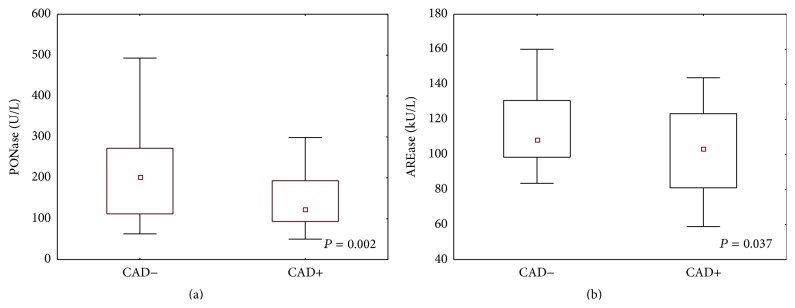
PONase activity (a) and AREase activity (b) in patients with coronary artery disease (CAD+) and patients with negative result of coronary angiography (CAD−). Values are presented as medians (25–75th percentiles, 5–95th percentiles) and assessed using the Mann-Whitney *U* test.

Patients with angiographically proven coronary artery disease, compared to patients with negative results, were characterized by significantly lower PONase activity (median with 25th and 75th percentiles: 123 (93–193) versus 201 (11–272)) and AREase activity (median with 25th and 75th percentiles: 103 (80–123) versus 109 (96–136)) (Figure 1).

**Figure 2:**
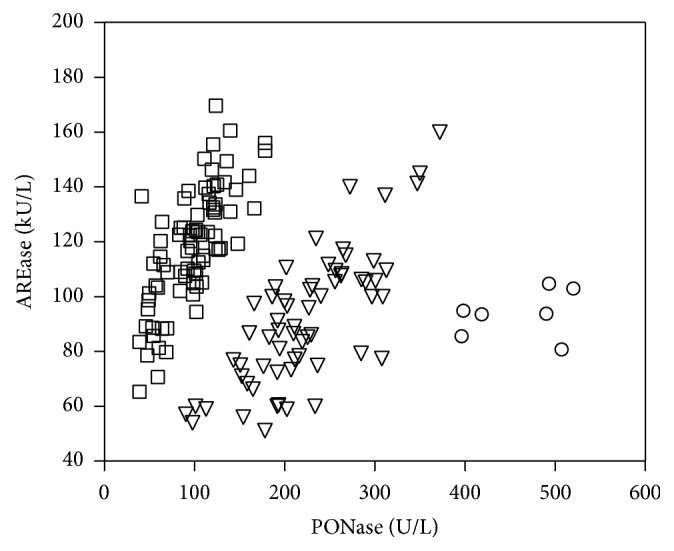
PONase activity towards AREase activity: squares: PONase/AREase < 1.5; triangles: PONase/AREase = 1.5–4.0; and dots: PONase/AREase > 4.0.

Figure 2 shows the distribution of paraoxonase (PONase) versus arylesterase (AREase) activity in all study populations. This relationship enabled the extraction of 3 groups (PON-1 phenotypes) of patients with different relative enzyme activity in the two different substrates (PONase versus AREase ratio less than 1.5, between 1.5 and 4.0, and greater than 4.0) as shown in Table 2. The percentages of phenotypes in CAD+ and CAD− participants were similar. In both groups, the PONase versus AREase ratio of most patients was less than 1.5; the smallest number of patients had a PONase/AREase value above 4 (Table 2).

**Figure 3:**
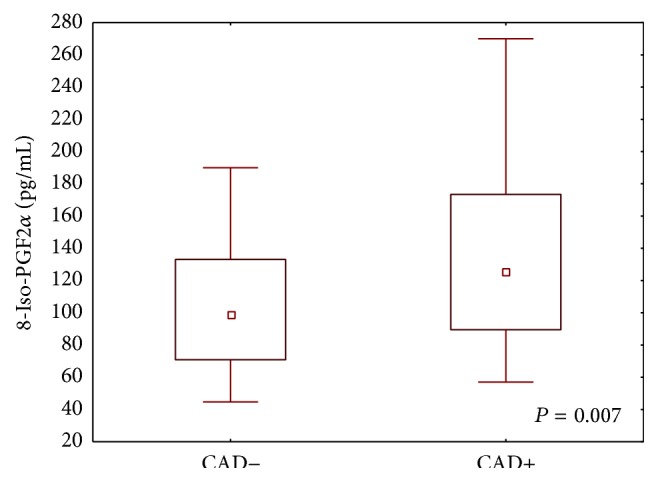
Plasma 8-iso-PGF2*α* concentrations in patients with coronary artery disease (CAD+) and patients with negative result of coronary angiography (CAD−). Values are presented as medians (25–75th percentiles, 5–95th percentiles) and assessed using the Mann-Whitney *U* test.

Patients with angiographically proven coronary artery disease had significantly higher concentrations of 8-iso-PGF2*α* compared to patients with negative results (median with 25th and 75th percentiles: 125 (89–173) versus 99 (71–133)) (Figure 3). There was no significant correlation between the activity of PON-1 and plasma 8-iso-PGF2*α* concentration in both the CAD+ patients and CAD− patients as also in the whole group of studied population. However evaluation of 8-iso-PGF2*α* concentration according to serum AREase and PONase activity quartiles had shown that the patients with lowest PONase activity have higher 8-iso-PGF2*α* level compared to the patients with the highest PONase activity (Table 3).

Analyzing the impact of traditional risk factor on the PON-1 activity and isoprostane levels we noticed the significant higher 8-iso-PGF2*α* level in CAD+ patients with hypertension compared to these without hypertension; similarly 8-iso-PGF2*α* levels were higher in smoking patients relative to nonsmoking patients. We did not observe the impact of diabetes on isoprostane concentrations. However diabetic patients had clearly reduced PONase activity compared to nondiabetic subjects (Table 4).

To evaluate coronary artery stenosis and determine the severity of CAD, we used angiography results drawn up on the basis of the Gensini score.

**Figure 4:**
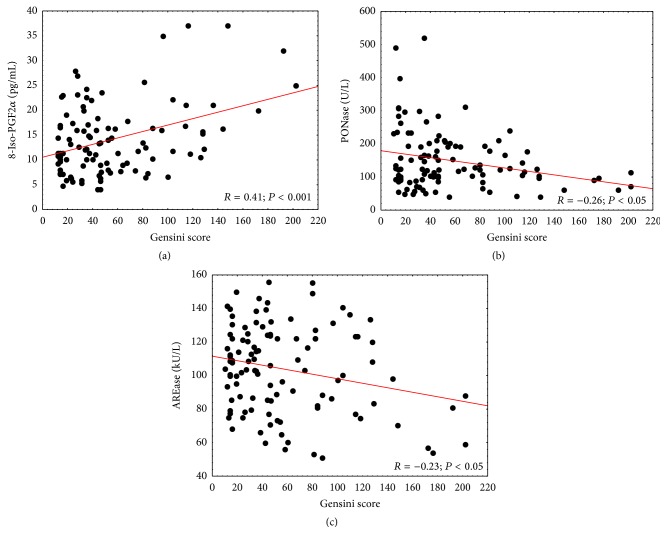
Correlations between Gensini score and (a) 8-iso-PGF2*α* concentrations, (b) PONase activity, and (c) AREase activity.

Univariate correlation analysis indicated that 8-iso-PGF2*α* concentrations were positively associated with Gensini scores (Figure 4(a)), whereas PONase and AREase activity were inversely correlated with the severity of CAD (Figures 4(b) and 4(c), resp.). 8-Iso-PGF2*α* concentrations and PONase activity remained an independent determinant of atherosclerosis severity in multiple linear regression after adjusting for age, gender, smoking habits, preexisting hypertension, diabetes, statin therapy, and HDL-C and TAG concentration (Table 5).

## 4. Discussion

The major finding of our study is the demonstration of decreased activity of PON-1 and increased concentration of 8-iso-prostaglandin F2 in patients with coronary artery disease (CAD) proved by angiography and establishment of the correlation between these factors and the severity of CAD.

Studies on PON-1 activity are based on quantifying its wide range of enzymatic activities in breaking down* in vitro* substrates such as paraoxon-paraoxonase (PONase) and phenyl acetate-arylesterase (AREase) activity. We have shown a significant reduction of PONase and AREase activity in a group of patients with angiographically proven coronary artery disease (CAD+) compared to patients with negative results of coronary angiography (CAD−) (Figure 1). The reduced activity of PON-1 is consistent with previous reports and confirms suggestions concerning the antiatherosclerotic properties of the enzyme [22, 23]. Moreover, our results show that the correlation between these two types of PON-1 activities is not simple [17]. The presentation of PON-1 activity results as a ratio of paraoxonase to arylesterase activities enables the extraction of three PON-1 phenotypes (Figure 2). The unique relationship between the two PON-1 activity measurements is probably the result of the stronger impact of genetic polymorphism on paraoxonase activity. A previous report has shown that the phenotype distinguished on the basis of the paraoxonase-to-arylesterase ratio closely corresponds to a common PON-1 polymorphism: Q (Glutamine) or R (Arginine) at codon 192 [15, 17, 18]. Several studies have examined the relationship between the PON 192 genotype and cardiovascular disease. Results are not consistent: whereas some have reported a correlation between the PON 192 allele and disease [24, 25], other studies have failed to find a connection, showing at the same time the decreased paraoxonase activity regardless of the PON-1 genotype [20, 26]. In our study, the percentage of phenotypes in the CAD+ and CAD− groups was similar (Table 2), which may support the hypothesis that the polymorphisms influence paraoxonase activity although this is clearly not crucial to the antiatherosclerotic properties of PON-1.

Apart from reduced PON-1 activity, CAD patients also were characterized by increased isoprostane levels (Figure 3). Isoprostanes are a class of biologically active products of arachidonic acid peroxidation that provides a reliable index of lipid peroxidation. Theoretically, a large number of isoprostanes types can be generated, but most interest has focused on F2 isoprostanes and in particular on 8-iso-prostaglandin F2 (8-iso-PGF2*α*). A previous study suggested increased concentrations of circulating 8-iso-PGF2*α* in coronary atherosclerosis development [27–30]. Clejan et al. suggested that accelerated isoprostane formation is intensified in CAD and predisposes patients to acute coronary syndrome [31]. Vassalle et al. showed that 8-iso-PGF2*α* concentrations are higher in CAD patients with multivessel disease compared to patients with single-vessel disease [32, 33]. Gross et al. showed an association between increased concentrations of circulating 8-iso-PGF2*α* and coronary calcification [30].

Our findings of elevated 8-iso-PGF2*α* levels in CAD+ patients are in agreement with earlier studies and support the hypothesis that an important role is played by the oxidative process in atherosclerosis.

Angiography, as the gold diagnosis standard for the coronary artery, is used not only to confirm the disease but also to assess the severity of atherosclerotic lesions using, among others, the Gensini score. One of the major discoveries of our study, after assessment of the severity of atherosclerosis using the Gensini score, was that 8-iso-PGF2*α* levels were positively, and PON-1 activity was negatively, correlated with the severity of coronary artery disease (Figure 4). Although, according to earlier reports [29, 34, 35], we have shown the impact of traditional risk factors for 8-iso-PGF2*α* concentration and activity of PON-1 (Table 4), PONase activity and 8-iso-PGF2*α* concentration remained independent determinants of atherosclerosis severity in multiple linear regression after adjusting age, gender, smoking habits, hypertension diabetes, statin therapy, and HDL-C and TAG concentration (Table 3). This confirms the hypothesis that the oxidative processes not only initiate the process of atherosclerosis but are also involved in the progression of the disease [23]. We failed to find simple correlation between paraoxonase activity and 8-iso-PGF2*α* concentration; however the patients with lowest paraoxonase activity have higher 8-iso-PGF2*α* level compared to the patients with the highest paraoxonase activity (Table 4), which may confirm, as previously suggested [36], possibility that these two opposing processes responsible for maintaining oxidant-antioxidant balance exert a reciprocal influence on one another.

Elevated levels of triglycerides and decreased levels of HDL are well-documented epidemiological risk factors for cardiovascular disease [37], which confirms the results of our work. However, many studies show that evaluation of HDL quality may have an impact on the prediction of CAD progression similar to the evaluation of numbers of HDL particles by measuring HDL cholesterol [38, 39]. In the development of atherosclerosis, HDL particles can lose their protective properties through changes in the oxidative processes or the loss of parts of molecules. The level of 8-iso-PGF2*α*, which assesses the severity of oxidant processes and the activity of PON-1, as an enzyme closely linked with HDL particles, can be valuable parameters for estimating the antiatherogenic properties of HDL.

Approximately 90% of the CAD+ study population had undergone statin therapy, which may explain the lower levels of LDL cholesterol and total cholesterol in patients with atherosclerosis compared to the CAD− group, in which 55% of patients had received statin drug therapy.

We did not observe a correlation between Gensini scores on one hand and LDL and total cholesterol level on the other hand. The impact of lipid-lowering therapy on the activity of PON-1 and 8-iso-PGF2*α* levels remains in question. Although some clinical studies showed that PON-1 activity increased and lipid peroxidation decreased in patients undergoing lipid-lowering therapy, no consensus has been reached [35, 40]. In our study, the small number of patients without statin therapy did not enable us to draw conclusions; however, patients with angiographically proven coronary artery disease, compared to patients with negative results of coronary angiography, were characterized by lower PON-1 activity and higher 8-iso-PGF2*α* concentration despite a higher percentage of statin users.

In summary, we have shown that oxidative stress plays an important role in the pathogenesis and development of CAD. The present finding suggests that impaired PONase activity and elevated 8-iso-PGF2*α* can be additional important biomarkers of coronary atherosclerosis development and in the future may be considered a potential pharmacological strategy for reducing CAD and preventing the progression of cardiovascular atherosclerosis.

## Figures and Tables

**Table 1:** Characteristics of patients with coronary artery disease (CAD+) and patients with negative result of coronary angiography (CAD−).

	CAD− *N* = 45	CAD+ *N* = 105	*P* value
Gender, M/F	20/25	41/64	0.537^*∗∗∗*^
Age (years)	63 ± 10	65 ± 10	0.252^*∗*^
BMI (kg/m^2^)	27 ± 4	28 ± 5	0.664^*∗*^
TAG (mg/dL)	102 (76–141)	107 (80–136)	0.882^*∗∗*^
TC (mg/dL)	196 ± 40	168 ± 41	<0.001^*∗*^
HDL-C (mg/dL)	52 ± 13	44 ± 11	<0.001^*∗*^
LDL-C (mg/dL)	125 ± 33	101 ± 37	<0.001^*∗*^
Apo AI (g/L)	1.7 ± 0.3	1.5 ± 0.3	<0.001^*∗*^
Apo B (g/L)	0.84 ± 0.17	0.79 ± 0.24	0.283^*∗*^
Apo B/Apo AI	0.52 ± 0.13	0.54 ± 0.18	0.512^*∗*^
Current smokers (%)	48%	66%	0.053^*∗∗∗*^
Diabetes (%)	22%	26%	0.566^*∗∗∗*^
Hypertension (%)	68%	79%	0.181
Metabolic syndrome (%)	42%	64%	0.143^*∗∗∗*^
Statin therapy (%)	55%	90%	<0.001^*∗∗∗*^

Values are presented as mean ± SD or as median (25th and 75th percentiles). ^*∗*^Student's *t*-test; ^*∗∗*^Mann-Whitney *U* test; ^*∗∗∗*^Pearson's chi-squared test.

**Table 2:** PON-1 phenotypes frequencies.

PONase/AREase	<1.5	1.5–4	>4
*N*	79	63	8
PONase/AREase	0.8 ± 0.2	2.5 ± 0.5	5.1 ± 0.7
Paraoxonase (PONase) [U/L]	98 ± 33	222 ± 62	465 ± 51
Arylesterase (AREase) [kU/L]	117 ± 22	92 ± 22	92 ± 12

CAD− (%)	42	47	11
CAD+ (%)	57	40	3

CAD+ patients with coronary artery disease and CAD− patients with negative result of coronary angiography; PONase and AREase activity are presented as mean ± SD.

**Table 3:** 8-Iso-PGF2*α* concentration according to serum arylesterase and paraoxonase activity quartiles.

	Paraoxonase (PONase) [U/L]				Arylesterase (AREase) [kU/L]			
	Quartile 1	Quartile 2	Quartile 3	Quartile 4	Quartile 1	Quartile 2	Quartile 3	Quartile 4
All subjects								
Range	<98	98–135	135–224	≥225	<87	87–107	107–122	≥123
8-Iso-PGF2*α* [pg/mL]	134 (130–196)	114 (89–157)	103 (74–146)	104^*∗*^ (69–154)	140 (100–200)	103 (70–162)	117 (74–148)	118 (83–168)
CAD+								
Range	<98	98–123	123–192	≥193	<83	83–103	103–123	≥123
8-Iso-PGF2*α* [pg/mL]	145 (88–158)	113 (78–161)	112 (88–158)	123 (84–169)	142 (103–205)	122 (89–184)	121 (75–157)	114 (76–168)
CAD−								
Range	<112	112–201	201–271	≥272	<98	98–108	108–130	≥130
8-Iso-PGF2*α* [pg/mL]	124 (70–170)	92 (49–125)	92 (74–130)	110 (71–137)	133 (80–153)	95 (69–133)	103 (74–119)	92 (72–149)

8-Iso-PGF2*α* concentrations are presented as median (25th and 75th percentiles); ^*∗*^ *P* < 0.05 compared with Quartile 1; CAD+ patients with coronary artery disease and CAD− patients with negative result of coronary angiography.

**Table 4:** Concentration of 8-iso-PGF2*α* and PONase and AREase activity in patients with and without traditional risk factors.

	8-Iso-PGF2*α* [pg/mL]	*P* value^*∗∗*^	Paraoxonase (PONase) [U/L]	*P* value^*∗∗*^	Arylesterase (AREase) [kU/L]	*P* value^*∗*^
Hypertension/nonhypertension						
All subjects	122 (80–170)/101 (79–137)	0.124	125 (93–202)/186 (104–234)	0.065	105 ± 26/112 ± 24	0.159
CAD+	134 (94–195)/101 (78–141)	0.045	121 (89–177)/152 (100–233)	0.501	102 ± 20/110 ± 26	0.218
CAD−	95 (69–138)/112 (80–133)	0.295	183 (99–272)/210 (201–311)	0.484	113 ± 23/114 ± 23	0.841
Diabetes/nondiabetes						
All subjects	120 (74–162)/116 (80–163)	0.894	104 (65–163)/153 (102–240)	<0.001	105 ± 25/108 ± 26	0.827
CAD+	126 (100–164)/120 (89–178)	0.694	104 (69–176)/125 (99–202)	0.049	102 ± 26/104 ± 23	0.761
CAD−	110 (71–119)/95 (72–135)	0.293	116 (64–140)/227 (127–306)	0.004	115 ± 25/115 ± 23	0.704
Smoking/nonsmoking						
All subjects	126 (89–178)/113 (70–137)	0.006	123 (93–201)/182 (109–264)	0.012	102 ± 26/112 ± 24	0.044
CAD+	140 (89–198)/116 (73–141)	0.042	131 (90–225)/119 (88–191)	0.074	99 ± 26/108 ± 24	0.153
CAD−	117 (90–138)/89 (66–133)	0.223	163 (96–238)/228 (140–300)	0.073	114 ± 20/117 ± 24	0.680

Values are presented as mean ± SD or as median (25th and 75th percentiles). ^*∗*^Student's *t*-test; ^*∗∗*^Mann-Whitney *U* test.

**Table 5:** Multiple linear regression analysis for Gensini score^a^.

	*β*	SE	*P* value
8-Iso-PGF2*α* ^*∗*^	0.368	0.09	<0.001
PONase^*∗*^	−0.267	0.10	0.01
AREase	−0.227	0.79	0.097

*β*: standardized beta coefficients; SE: standard error; ^a^adjusted for age, gender, smoking habits, hypertension, type 2 diabetes, statin therapy, and HDL-C and TAG concentration; ^*∗*^log-transformed values.
